# Measurable Indicators of CRPD for People with Intellectual and Developmental Disabilities within the Quality of Life Framework

**DOI:** 10.3390/ijerph17145123

**Published:** 2020-07-15

**Authors:** Laura E. Gómez, Asunción Monsalve, Mª Lucía Morán, Mª Ángeles Alcedo, Marco Lombardi, Robert L. Schalock

**Affiliations:** 1 Department of Psychology, Universidad de Oviedo, 33003 Oviedo, Spain; monsalve@uniovi.es (A.M.); moranlucia@uniovi.es (M.L.M.); malcedo@uniovi.es (M.Á.A.); 2 E-QUAL, University College Ghent, 9000 Gent, Belgium; marco.lombardi@hogent.be; 3 Department of Psychology, Hastings College, Hastings, NE 68901, USA; rschalock@ultraplix.com

**Keywords:** rights, CRPD, intellectual disability, assessment, indicators, quality of life, convention, developmental disabilities, personal outcomes, PRISMA

## Abstract

This article proposes the quality of life (QOL) construct as a framework from which to develop useful indicators to operationalize, measure, and implement the Articles of the Convention on the Rights of Persons with Disabilities (CRPD). A systematic review of the scientific literature on people with intellectual and developmental disabilities (IDD) was carried out, with the aim of identifying personal outcomes that can be translated into specific and measurable items for each of the CRPD Articles aligned to the eight QOL domains. Following Preferred Reporting Items for Systematic Reviews and Meta-Analyses (PRISMA) guidelines, the systematic review was conducted across the Web of Science Core Collection, Current Contents Connect (CCC), MEDLINE, KCI-Korean Journal Database, Russian Science Citation Index and SciELO Citation Index, for articles published between 2008 and 2020. A total of 65 articles focusing on people with IDD were selected. The results were grouped into four broad categories: conceptual frameworks used to monitor the CRPD; instruments used to assess the rights set out in the CRPD; recommendations on the use of inclusive research; and indicators or personal outcomes associated with specific rights contained in the CRPD.

## 1. Introduction

Changes in how people with intellectual disabilities and developmental disabilities (IDD) are perceived, and prevailing attitudes toward them, are increasingly reflected not only in national laws and regulations, but also in specific international conventions currently being used worldwide to develop, implement, and monitor social policies and professional practices aimed at promoting the inclusion and independence of people with ID in society [1,2,3].

More than a decade ago, in an effort to focus attention on the dignity of people with disabilities and their right to participate fully in community life in the same way as any other citizen, the United Nations Convention on the Rights of Persons with Disabilities (CRPD) was approved and ratified by 180 countries [4]. The CRPD was an international milestone recognizing the change in attitudes toward people with disabilities, based on the premise that people with disabilities, including those with ID, should have an active role in making decisions about their own lives, carry out productive activities, be included in society, and receive appropriate supports to allow them to live as full citizens, on an equal basis with others. Thus, the CRPD sets out rights that go far beyond what is strictly required by law, emphasizing economic, social, and cultural rights [3,5]. In its ambition to be more than a mere declaration of principles, the CRPD also underlines the importance of collecting data to assess the extent to which people with disabilities perceive that their rights are being respected and implemented [6].

The CRPD contains 50 Articles. Articles 1 to 4 set out the purpose of the Convention (Art. 1), define the concepts used (Art. 2), and outline the principles on which the CRPD is based (Art. 3) and the general obligations of States Parties (Art. 4). Articles 5 to 30 (i.e., a total of 26) then detail specific obligations for States. These 26 Articles defend the right of persons with disabilities (including those with ID) to enjoy legal capacity in all aspects of life and on an equal basis with others (Art. 12: equal recognition before the law); to have the opportunity to choose their place of residence, where and with whom they live, without being obliged to live in a particular living arrangement (Art. 19: right to live independently and to be included in the community); to access, on an equal basis with others, the physical environment, transportation, information and communications (Art. 9: accessibility); to access information in accessible formats and with technologies appropriate to different types of disability in a timely manner and without additional cost (Art. 21: freedom of expression and opinion, and access to information); to marry and found a family on the basis of free and full consent (Art. 23: respect for the home and the family); not to be excluded from the general education system, ensuring the reasonable accommodation of the individual’s requirements, as well as the provision of the necessary individualized support measures to facilitate their effective education within the general education system (Art. 24: education); to access the same range, quality and standard of free or affordable health services as provided to other persons, as close as possible to their communities, including in rural areas (Art. 25: health); to have at their disposal comprehensive habilitation and rehabilitation services and programs in the fields of health, employment, education and social services, available from the earliest possible stage, based on their needs and strengths, and provided by suitably trained professionals (Art. 26: habilitation and rehabilitation); to have the opportunity to earn a living through work freely chosen or accepted in a labor market or work environment that is open, inclusive, and accessible (Art. 27: work and employment); and to participate fully and effectively in political and public life on an equal basis with others, directly or through freely elected representatives (Art. 29: participation in political and public life). Following these 26 Articles containing specific rights, the next 10 (Arts 31–40) deal with data collection and statistics (Art. 31); international cooperation (Art. 32); national implementation and monitoring of the CRPD (Art. 33); the establishment and functioning of the Committee (Art. 34); reports (Arts 35, 36 and 39); the relationship between the Committee, States and other bodies (Arts 37 and 38) and the regular conference with States Parties (Art. 40). Finally, the last 10 Articles (Arts 41–50) are reserved for signature, depositary, entry into force and other similar issues.

Implementing the CRPD is not without its difficulties. The abstract nature of rights and their context-based expression pose a challenge for evaluation and implementation [1,2,7,8], making it essential to define specific targets and objective indicators that can be used to assess progress [9,10]. Because of the significant conceptual and measurement work done in the field of quality of life (QOL) several authors have suggested that the QOL construct provides a valid framework from which to develop useful indicators to operationalize, measure, and implement the CRPD Articles (1,2,11), and to ultimately assist organization and systems transformation. The QOL construct provides an ideal conceptual framework for evaluating rights-related personal outcomes [11,12,13] and for translating abstract political concepts —such as self-determination, equity, accessibility, or inclusion [1,2,11,14]—into evidence-based practices [15,16,17].

This article proposes the QOL conceptual framework as a means of evaluating and implementing the CRPD Articles with respect to people with intellectual and developmental disabilities (IDD). The ultimate aim of the purpose of using the QOL framework is not to provide precise statistics on rights violations for the reports submitted to the United Nations Committee, but rather to: (a) give a voice to people with ID regarding everyday situations in their daily lives that are frequently not measured using conventional evaluation methods; (b) serve as a tool that professionals and relatives can use to detect any breach, abuse or denial of rights, thereby helping them enhance the supports they provide to people with ID; and (c) provide guidance to organizations on the strengths and greatest needs of people with ID in relation to rights that are encompassed in the CRPD. In this way, the approach to the evaluation of rights set out in this article focuses more on the microsystem (i.e., improving the lives of people with ID) and on the mesosystem (i.e., improving the provision of generic and professional supports offered by organizations) than on the macrosystem (i.e., lawmaking or production of official national statistics).

Several conceptual models of the QOL construct exist in the field of ID [18,19,20], although all of them highlight four basic principles in their definition [16]: (a) QOL is composed of several dimensions (i.e., multidimensional) that are the same for all people (i.e., universality); (b) it is influenced by personal and environmental factors; (c) it has both objective and subjective components; and (d) it is enhanced by individualized, person-centered supports. In this article, we focus on the QOL model proposed by Schalock and Verdugo [20]. It is the most widely accepted and cross-culturally validated model of QOL, and is used widely internationally by ID support organizations and systems in support provision, organization transformation, and systems change. [21,22,23,24,25,26,27]. According to Schalock and Verdugo’s conceptual framework, QOL encompasses eight core domains that interact with each other. These domains include rights, self-determination, social inclusion, interpersonal relationships, personal development, emotional wellbeing, material wellbeing, and physical wellbeing. The eight domains can serve as a basis to evaluate the implementation of the 26 CRPD Articles on specific rights, through the measurement of core indicators and personal outcomes associated with QOL.

Two pioneering studies have sought to operationalize the CRPD through the QOL conceptual framework. The first demonstrated the theoretical foundations of the close relationship between the 26 CRPD Articles and the eight QOL domains, and put forward an initial proposal to organize the Articles by QOL domain [1]. The second article reported on the international consensus on the relationship between the core QOL indicators and the CRPD Articles [2]. To this end, a Delphi study was conducted with 153 experts (comprising people with ID, family members, professionals, researchers, and law experts) from 10 countries (Brazil, Canada, the Czech Republic, Germany, Israel, Italy, Portugal, Spain, Taiwan, and the United States). Consensus was reached on over 80 cross-culturally agreed and validated QOL indicators, which were aligned to the eight QOL domains.

Results from both of these studies are summarized in Table 1. They represent the framework for the systematic review presented in this article. The overall aim of this review is to take the next logical step in the operationalization of the CRPD: to review the scientific literature on people with IDD, in order to identify indicators and personal outcomes that can be translated into specific and measurable items for each of the CRPD Articles that are aligned to the eight QOL domains. In particular, the present systematic review sought to answer the following questions:

- What is discussed in the publications about the CRPD and people with IDD?
- In the literature, what theoretical frameworks and assessment instruments are proposed to monitor the implementation of the CRPD for people with IDD?
- What indicators or personal outcomes are mentioned in the scientific literature discussing the CRPD Articles that protect specific rights for people with IDD?

## 2. Materials and Methods

### 2.1. Inclusion and Exclusion Criteria

The following inclusion criteria were applied for this systematic review of the scientific literature. First, publications had to be peer-reviewed articles published since 2008, when the CRPD came into force. Thus, articles published between 2008 and January 15, 2020 were included. Second, studies had to refer to the CRPD and to people with IDD. Third, articles had to be published in English or in Spanish.

Given that one of the aims of this research was to locate as many indicators and personal outcomes as possible, added to the fact that the specific nature of the search was unlikely to return a large volume of results, few exclusion criteria were predefined. Following an examination of the complete references, results that had initially met the inclusion criteria set out above were subsequently excluded if they: (a) did not focus on the CRPD; (b) did not include people with IDD; (c) were letters, editorials, books, book chapters, indexes, and proceedings; or (d) were legal texts (i.e., documents limited to describing or analyzing regulations, laws, and conventions).

Articles that met this initial screening were retrieved and read in full, and were subsequently excluded if they: (a) were not related to the CRPD; or (b) did not refer to indicators or personal outcomes associated with any of the 26 Articles pertaining to specific rights (Table 1) for people with IDD.

### 2.2. Search Procedures

Two separate searches were conducted in parallel: a search of the scientific literature in English and another of the scientific literature in Spanish. The search terms were chosen based on the individual English-language and Spanish-language contexts. In other words, no attempt was made to produce a literal translation of the search terms from English into Spanish or vice versa, but the aim was rather to reproduce the meaning of the search terms and expressions taking into consideration possible cultural differences. For example, the term “learning disabilities” in Spanish is not used as in other countries to refer to people with “intellectual disabilities”, but instead refers to people with specific learning disorders, for example, in reading, writing, or solving mathematical problems.

In order to identify studies on the CRPD and people with ID published in English, a systematic review was carried out on Web of Science (WOS). The databases included in the search were Web of Science Core Collection, Current Contents Connect (CCC), MEDLINE, KCI-Korean Journal Database, Russian Science Citation Index and SciELO Citation Index, incorporating the publication timespan 2008–2020 as an inclusion criterion. The search terms used in the TOPIC field were: “Convention” *OR* “CRPD” *OR* “UNCRPD”, combined with *AND* “intellectual disability *” *OR* “developmental disability *” *OR* “intellectual developmental disorder *” *OR* “learning disability *”. Table 2 summarizes the exact WOS search, which returned a total of 200 publications.

For the search in Spanish, the two platforms of Scopus and ProQuest were used, covering nine databases: Bibliografía de la Literatura Española, EconLit, Literature Online, Philosopher’s Index, PsycARTICLES, PsycINFO, PsycTESTS, PTSDpubs and Publicly Available Content Database. In both platforms, the search criteria were set to include papers published in Spanish between the years 2008 and 2020. Furthermore, the search in ProQuest was limited to “peer-reviewed” documents. In both databases, the Spanish-language search terms were “Convención” *OR* “CDPD” *OR* “CDPCD”, combined with *AND* “discapacidad intelectual” *OR* “discapacidad * del desarrollo” *OR* “trastorno * del desarrollo” in any field in ProQuest and in the “Article title, Abstract, Keywords” fields in Scopus. The Spanish search yielded a total of 53 results (Table 2), giving a combined search total of 253 publications across the two languages.

### 2.3. Article Selection

Data from the 253 articles identified across the three search platforms were incorporated into one Excel database. After four duplicates were removed—leaving a total of 249 articles —references (title, abstract, publication title, and pages) were screened by the first author of this paper for alignment with the inclusion and the exclusion criteria outlined above. This reference screening phase reduced the initial pool of documents to 136. Articles were removed at this stage primarily because they were not related to the CRPD (44.2%), they were not articles (18.6%), or they were limited to legal aspects (21.2%). A further 12.4% were not written in English or Spanish, and 3.5% did not refer to or include people with IDD. Next, 30% (*n* = 75) of the results were randomly selected and reviewed by the third author in order to examine the reliability of the decisions about inclusion or exclusion. The level of agreement between the two researchers was 89.3% in the first round and was 100% with regard to the reason for excluding a document. The first four authors of this article discussed the eight papers for which there initially had been disagreement and reached a consensus for all of them.

**Figure 1:**
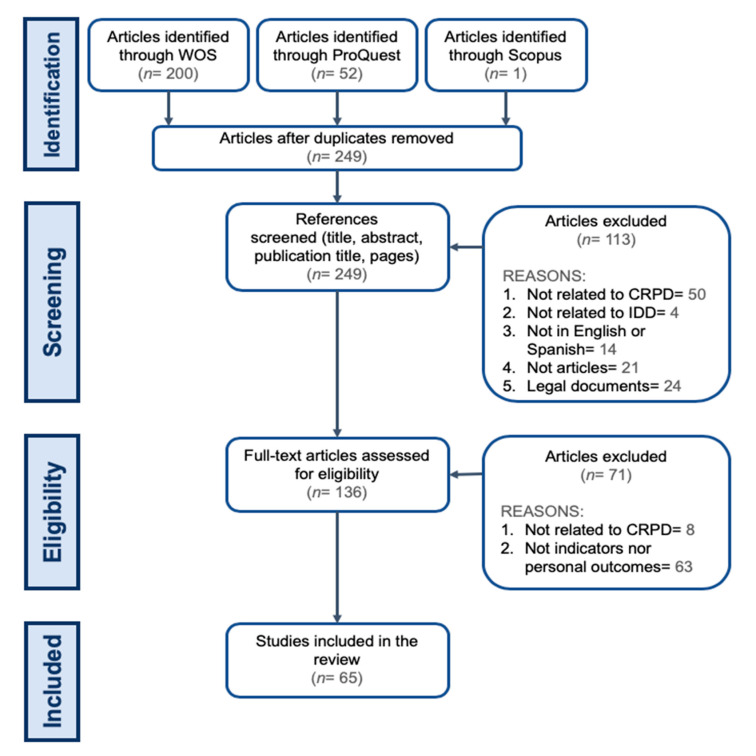
Search and article selection flow diagram.

The next step was to retrieve the full-text versions of the 136 selected documents and to assess them for eligibility. After reviewing the full texts, 71 results were excluded because they were not related to the CRPD (11.3%) or because they did not refer to explicit indicators or personal outcomes related to specific rights set out in the CRPD (88.7%). Replicating the process described above, 30% (*n* = 41) of the full-text documents were randomly selected and reviewed, this time by both the first and the third authors of this paper. In the first round, the decision to exclude an article or not on the basis of the two reasons outlined above obtained an inter-rater agreement of 80.5%. The first four authors then discussed the eight papers for which there had been disagreement, and a consensus was ultimately reached. As a result, 65 articles were considered to have met the inclusion criteria (marked with * in References) and form the pool of documents upon which the results of this review are based. The entire process is illustrated in Figure 1.

### 2.4. Article Coding and Data Extraction

First, the 65 articles selected for inclusion in this systematic review were grouped into four broad categories: (1) articles that refer to conceptual frameworks (e.g., QOL models) for CRPD monitoring (i.e., they relate to Article 31 “Statistics and data collection” or Article 35 “Reports by States Parties”); (2) articles that propose or use instruments to assess the rights contained in the CRPD (for a specific Article or for several of the Articles from 1 to 50); (3) articles that use or discuss inclusive research (given the solid foundation for ethical, inclusive research with people with disabilities provided for by the CRPD, and particularly the explicit mention in Article 33 “National implementation and monitoring” that “persons with disabilities and their representative organizations must be involved and participate fully in the monitoring process”); and (4) articles that include indicators or personal outcomes associated with one or more of the 26 Articles pertaining to specific rights (i.e., Articles 5 to 30 of the CRPD). Articles assigned to the third category—inclusive research—could simultaneously be classified into one of the other three categories. Categories 1, 2 and 4, on the other hand, were considered mutually exclusive for article coding and categories 1 and 2 were prioritized over category 4. None of the articles could be classified in category 1 and 2 at the same time, but a few could be classified in categories 1 and 4, or categories 2 and 4. In these cases, they were assigned to categories 1 and 2, respectively, but they were also scrutinized in order to identify indicators or personal outcomes.

Secondly, all the articles were coded and subgrouped according to the specific CRPD Article number they referred to. Finally, all articles were classified depending on whether they were: (1) quantitative—descriptive papers; (2) qualitative—descriptive papers; (3) mixed methodology; (4) reviews; (5) position papers; or (6) descriptions or proposals for interventions, programs or practices.

Similar to the process used to test reliability at the article exclusion step, the inter-rater reliability was established for the coding criteria set out above. The first author coded 100% of the articles, and the third author coded 30% (*n* = 20). In the first round, the inter-rater agreement regarding the decision on category (i.e., framework, instrument, specific Article, or inclusive research) was 95% (*n* = 1 disagreement); for the CRPD Article number it was 85% (*n* = 3 partial disagreements; there was total agreement on the main Article being referred to but some disagreement when there were several secondary Articles mentioned); and, finally, for the type of document (i.e., quantitative, qualitative, review, position, intervention), the inter-rater agreement was 85% (*n* = 3 disagreements). All disagreements were resolved by a discussion among the first four authors and a consensus was reached.

## 3. Results

A total of 65 articles published between 2008 and 2019 were included in this review. Only four articles were identified for the publication years 2008–2010, with most published after 2017 (*n* = 31; Me = 2016), and the highest publication output recorded in 2019 (*n* = 13). In all, 89.2% of the articles were indexed in WOS, 9.2% in ProQuest, and 1.5% in Scopus. Most of the papers were written in English (86.15%), while only nine were written in Spanish.

These publications involved a total of 348 authors (or 411 including duplicates, where the same author has written more than one paper). With four publications each, the most prolific authors on this subject are J. Fullana, E. García, P. McCallion, M. McCarron, M. Pallisera, and M.A. Verdugo; followed by T. Carney, G. Díaz, L.E. Gómez, M. Redley, N. Salmon, R.L. Schalock, and M. Vila, who have three publications each. The 65 papers were published across a wide variety of journals (*n* = 37), with the largest concentration in the Journal of Policy and Practice in Intellectual Disabilities (*n* = 12), Journal of Intellectual Disability Research (*n* = 6), Journal of Applied Research in Intellectual Disabilities (*n* = 4), British Journal of Learning Disabilities (*n* = 4), Disability and Rehabilitation (*n* = 3), Tizard Learning Disability Review (*n* = 3), International Journal of Law and Psychiatry (*n* = 3), and Disability & Society (*n* = 2). Only one manuscript was published in each the remaining journals.

With regard to the geographical regions covered by the studies, more than half were conducted in Europe (53.8%), and one-quarter each in Oceania (26.2%) and North America (23.1%). There is a lower volume of studies conducted in—or referring to—Asia (10.8%), South America (9.2%), and Africa (6.2%), while only one was carried out in the Middle East (i.e., Israel; 1.5%). Out of a total of 34 countries, the highest number of studies was carried out in Australia (*n* = 16), Spain (*n* = 13), Ireland (*n* = 11), Canada (*n* = 8), England (*n* = 6), and the United States (*n* = 6).

### 3.1. What is Discussed in the Publications about the CRPD and People with IDD?

The vast majority of the publications (*n* = 48; 73.8%) focused on one or more of the 26 CRPD Articles pertaining to specific rights (i.e., Articles 5 to 30). Only eight papers (12.3%) addressed conceptual frameworks for CRPD monitoring (i.e., they relate to Article 31 “Statistics and data collection” or Article 35 “Reports by States Parties”), and five (7.7%) proposed or applied assessment instruments (for a specific Article or for several of the Articles from 1 to 50). A total of eight (12.3%) used or discussed inclusive research (Article 33).

Most were descriptive studies (*n* = 34)—qualitative (29.2%), quantitative (18.5%), or mixed methodology (4.6%)—and theoretical articles or position papers (27.7%), while 11 were reviews (19.9%), and only four described interventions, programs or practices (6.2%). All referred to people with IDD, although almost one-fifth (*n* = 12; 18.5%) discussed people with disabilities in general, while 6.2% referred specifically to people with Down syndrome (*n* = 2), cerebral palsy (*n* = 1), or neurologic conditions (*n* = 1).

### 3.2. In the Literature, What Theoretical Frameworks and Assessment Instruments are Proposed to Monitor the Implementation of the CRPD for People with IDD?

Of the eight papers that used or proposed conceptual frameworks to monitor or evaluate the CRPD or its specific Articles, all pointed to the QOL framework and QOL indicators that must be incorporated into comprehensive instruments to assess progress and identify needs and gaps in implementation. The QOL model proposed by Schalock and Verdugo was the most commonly used [1,2,8,9,11]—although the Cummins model [28] is also mentioned—and applications in specific contexts—namely “Educational quality of life” [29]—are also discussed. As shown in Table 3, five of the studies named, applied or proposed specific measurement tools to assess QOL (QOL-Q, Integral Scale, Gencat Scale, Personal Outcomes Scale, Personal Wellbeing Index), self-determination (AIR Self-Determination Scale), or rights (Rights of Persons with Disabilities ad hoc scale). Only one study used qualitative instruments in the form of focus groups and in-depth interviews [30]. While all of these papers referred to a QOL conceptual framework as a way of evaluating or monitoring the CRPD (Articles 31 and 35), some also focused on other CRPD Articles, the most common being (*n* = 2) Article 7 (children with disabilities), Article 19 (living independently and being included in the community), and Article 24 (education). In the eight studies, the countries where this framework was most used or proposed were Australia, the United States, and Spain.

In the five studies that used or proposed specific tools to monitor compliance with the CRPD, a total of five instruments are cited (Table 4). None of them were originally designed with the specific objective of monitoring the CRPD, although the structure of the ITINERIS Scale [31]—while inspired by the Montreal Declaration—was developed by five professionals who independently evaluated the relationship between the items in the scale and the preamble and first 30 Articles of the CRPD. The four other instruments proposed to monitor certain Articles of the CRPD are the rights subscales of QOL scales, comprising the Gencat Scale or the Integral Scale [13]; the National Core Indicators-Adult Consumer Survey (NCI-ACS), aimed at assessing the quality of services [3,32]; and the European Child Environment Questionnaire (ECEQ), which covers physical, social and attitudinal environmental features [33]. The number of items in these five instruments ranges from 8 to 51, and all are designed for adults with ID, save for the ECEQ, which was developed for children with cerebral palsy. In addition, most of the instruments are self-reports administered directly to people with disabilities, while only two are other-reports answered by relatives or professionals. Of the specific CRPD Articles assessed, the range covered by the instruments is limited to the first 30. Articles 21 (freedom of expression and opinion, and access to information) and 22 (respect for privacy) are dealt with in most of the instruments (*n* = 4), followed by (*n* = 3) Articles 3 (general principles), 9 (accessibility), 18 (liberty of movement and nationality), 23 (respect for home and the family), and 27 (work and employment).

Finally, the sociodemographic data gathered in these studies includes the following: (a) personal variables such as gender, age, ethnicity, language, religion, sexual orientation, level of ID, cause of ID, disability onset, civil status, educational level, need of assistive products, mental health diagnosis, problematic behavior, verbal expression, sensory impairment, support needs, residential type, mobility, socioeconomic status, guardianship, advocacy experience; (b) contextual variables such as region, degree of urbanicity, community size, level of involvement and frequency of contact with family or professionals, care and residential setting, type of school setting, employment.

It is also important to mention the eight studies that focus on inclusive research, the majority of which were conducted in Ireland (*n* = 5), the others in Spain (*n* = 2) and Australia (*n* = 1). The inclusive research model holds that people with disabilities with relevant experience related to the studied topic should be included in the research, not only as informants but participating and making decisions in all phases. This level of involvement is essential in order to comply with the part of Article 33 where it states that “civil society, in particular persons with disabilities and their representative organizations, shall be involved and participate fully in the monitoring process”. Inclusive research incorporates a wide range of research approaches that have traditionally been referred to as “participative”, “action”, or “emancipatory”. Some authors have tried to differentiate between the techniques, arguing that the emancipatory approach is achieved when the initiative and research topic is proposed by people with disabilities, and it is they who control the research, while the participative approach places greater emphasis on the representation of people with disabilities at all stages of the research process [34,35]. The most common ways for people with ID to participate in these studies are by providing their views and opinions through focus groups, structured interviews, and workshops [35,36,37,38]; by acting not only as study participants, but also as advisory committee members and co-researchers [39,40], making it essential that they receive research training [41]. The most frequently cited limitations allude to the challenge of remaining inclusive throughout data collection and analysis, together with including people with complex support needs, such as those with profound and multiple disabilities or who use alternative or augmentative communication approaches.

### 3.3. What Indicators or Personal Outcomes Are Mentioned in the Scientific Literature Discussing the CRPD Articles that Protect Specific Rights for People with IDD?

The indicators and personal outcomes identified in the 48 studies that deal with the specific rights in the CRPD (Arts 5–30) are aligned to the eight QOL domains and presented below.

#### 3.3.1. Personal Development

- Article 24 (education): Nine articles included in this review refer to specific indicators and personal outcomes related to inclusive education [8,42,43,44,45,46,47,48,49] in ordinary settings at all levels of education (preschool, primary, secondary, high school, vocational training, university). The specific indicators mentioned were the right to attend educational establishments near their community; individualized supports within the general education system; assessment of individual support needs in environments that maximize academic and social development; completion of stages and appropriate transitions between them; coordination among the different professionals involved; training about rights; training about sexuality, reproduction and family planning (understanding what sexual relationships are, risks, benefits and alternatives; questions about sexuality can be freely raised and resolved); training and preparation for independent living (in real-life contexts, from compulsory education); vocational guidance; adequate training and qualifications to get a job; individualized educational aids (e.g., teacher’s aide, tutors, extended test time, modified course curriculum); appropriate materials; reasonable accommodations; quick access to necessary educational support products (e.g., specialized software, recording or note taking devices, audio/e-book devices); information, care, and guidance services for families (e.g., legislative measures and supports related to the education of their children); participation of the family in the education process; information, care, and guidance services for teachers about disability, supports and special educational needs; attitudes toward the inclusion of family members and teachers; meaningful learning experiences; participation in the activities of the school; the school and its staff enhance the person’s self-esteem, satisfaction, autonomy, and self-confidence; friends at school (not only among staff or carers); educational institutions in a holistic perspective of health and care.

#### 3.3.2. Self-Determination

- Article 14 (liberty and security of person): 10 papers [8,38,40,45,48,49,50,51,52,53] address this Article, specifically freedom of choice (e.g., to choose where and with whom they live, the type of housing, moving house, what to cook, how to spend their free time and with whom); making their own decisions (including decisions about health); personal autonomy (e.g., control of their finances, handling their own money independently, not being overprotected by their family, not being underestimated by their parents or still being perceived as a child); control over life and life events (e.g., social outings, simple events in their daily lives); upbringing experiences focused on developing skills for independence and self-determined behaviors; coping strategies (e.g., impact of health problems on daily life); person-centered approach.
- Article 21 (freedom of expression and opinion, access to information): Seven studies focus on access to—and understanding of—information, as well as opportunities to use information and express their opinion [8,40,48,51,54,55,56]. From these, the following personal outcomes were extracted: information in accessible formats (e.g., easy-read format); assistive products for communication and cognition (knowledge and awareness; customization); access to the internet, its content and digital services (e.g., adapted applications, internet sites and web browsers; modifying the mouse settings or enlarging the font); technological devices adapted to the person’s specific needs (e.g., alternative mice, enlarged keyboards, touch screens, voice synthesis and recognition systems); technical support (e.g., configuring device security, securing the wireless network, installing an antivirus program, setting up the firewall, updating the operating system and software); participation in digital society (educational programs, individualized supports to understand new social interaction rules and conventions); self-advocacy (to have their voice heard, confidence to speak up, defend their health, sexual and emotional options).

#### 3.3.3. Interpersonal Relationships

- Article 23 (respect for home and the family): 10 articles included in the review [8,39,40,49,57,58,59,60,61,62] refer to the right to have opportunities to meet people, establishing relationships, having friends, meeting the right person, and having a partner relationship (taking risks to be with the person they want, opposing control of the family and restrictive service regulations, receiving support without being treated as children or considered asexual or unable to raise children, choosing their sexual orientation, being listened to about their needs); to marry and found a family (retain their fertility on an equal basis with others, avoiding forced sterilization and covert contraception, making their own reproductive and sexual choices, deciding on the number of children to have); to keep their own children with them (i.e., receiving specific supports for the wellbeing of both children and parents with disabilities, obtaining legal custody of the children in case of divorce, avoiding the separation of children from their parents against their will on the basis of a disability, or denial of their rights as fathers/mothers); to receive sexual information, guidance, and support in caring for their children (e.g., basic care, nutrition, health, education; home-based learning and flexible support services over the long-term that are evidence-based, tailored to individual needs, and built on the strengths of each parent and family; monitoring of new needs); to be able to adopt and foster, and to access assisted reproduction; guidance and training for families and professionals who provide evidence-based methods and non-discriminatory support in sexuality (preventing negative attitudes of professionals and families toward sexuality, such as discouraging marriage and parenting, restricting sexual expression; disapproving relationships, allowing platonic but not intimate relationships); organization policy facilitating sexual experiences and comprehensive sex education programs (not only addressing biological facts but also allowing them to discuss the social and emotional aspects of relationships and sexuality, to learn about abuse and exploitation, to recognize the importance of desire and pleasure).

#### 3.3.4. Social Inclusion

- Article 8 (awareness-raising): Seven articles [8,48,59,63,64,65,66] provide indicators and personal outcomes related to advertising campaigns. The specific personal outcomes include giving them visibility; promoting normalization and generating awareness about disabilities; treating their image with respect; sensitizing and ensuring the realization of their rights; promoting equality; improving participation and inclusion; breaking stereotypes. In addition, there was an emphasis on the need for specific awareness campaigns focusing on the reality of women with ID and their capability of parenting (associated with Articles 6 and 23, respectively).
- Article 9 (accessibility): Four studies [46,48,53,67] mention specific elements that could be operationalized with respect to the accessibility of the environment. In particular, they refer to accessibility in health facilities, community centers, educational establishments, and workplaces; accessibility in public infrastructure; accessibility of leisure environments.
- Article 18 (liberty of movement and nationality): Only one article [40] discusses the freedom to use public transport; freedom to move around and have control over their movements; being within walking distance of amenities and shops.
- Article 19 (living independently and being included in the community): 14 papers [8,38,39,40,42,45,47,49,50,53,63,66,68,69] mention personal outcomes or indicators associated with the right to live independently and be included in the community (i.e., not to be institutionalized in segregated environments, not to be restricted in options for in-home residential and other community support services). To achieve this, the papers highlighted the right to receive the necessary individualized supports (person-centered planning, individualized support to live more independently) for everyday activities to do with autonomy in the home (e.g., getting to appointments, running errands, housework, personal finances, heavy household chores, preparing meals, personal care and medical care); support from professionals (sufficient personal resources) and service providers (to organize preferred housing, help find housemates, forge social connections); in housing, to facilitate flexibility in terms of rules and staff control, freedom to move around and to arrange daily home life, enjoy own space, individualized care, small groups, living with their partner; trust and support from family (role of the family as a source of support and as facilitators of autonomy, opportunities to practice skills, avoiding overprotection); special attention to access supports during and after moving (to organize move, types of support, relationships with supporters, quality of supporters); being within walking distance of amenities and shops; support for older people with disability; control over support arrangements (choose support workers and the kind of support they receive); housing affordability; access to information on independent living experiences.
- Article 20 (personal mobility): Two articles [54,63] include specific aspects related to this right. The indicators and personal outcomes mentioned are a way to be personally mobile (availability of assistive products for mobility; knowledge and awareness about them; customization); and a way to transport across environments (i.e., adapted transport; human support and vehicle available).
- Article 29 (participation in political and public life): Seven studies [8,49,53,66,70,71,72] consider aspects related to the right of people with ID to vote (e.g., information about the meaning and content of elections and democratic participation; understanding the information from the parties, the electoral procedures, and the voting paper; easy postal votes; accessible local polling stations; using pictures, symbols and logos on the voting paper; courses about voting and elections; easier-to-read election materials; support at polling stations and during the process of voting; treated respectfully by election officials; web accessible guides to voting); to be elected political members; priority on supportive legislation on disability issues in the government and CRPD focus.
- Article 30 (participation in cultural life, recreation, leisure and sport): Measurable indicators and outcomes are found in eight articles [40,47,48,49,50,53,66,69]. They refer to being part of society, taking part in all aspects of their community life; having sufficient income to participate in the community; adequate information about community activities for families (e.g., organized social leisure activities); participating in activities with people without ID (e.g., inclusive sports); promotion of active participation and play in their communities; enjoying leisure time (doing a variety of things alone or with others; relaxing and having fun; doing things they enjoy; going to pubs and night life; not being ignored at social events); awareness, positive attitudes and actions of other members of the community.

#### 3.3.5. Rights

- Article 5 (equality and non-discrimination): Five papers [49,50,54,69,73] identify the indicators and personal outcomes of not suffering stigma; not suffering discrimination (e.g., in insurance matters, in access to health care); not experiencing rejection and denial of their individuality, adulthood and capacity.
- Article 6 (women with disabilities): Eight papers refer to the rights of women [8,49,52,58,59,60,65,74]. Most indicators and personal outcomes were already reflected in other CRPD rights, but there was particular emphasis on the application of these rights—and respect for—women. The most frequently cited aspects in relation to women include prevention and intervention in gender-based violence (knowing their rights, knowing how to deal with acts of aggression perpetrated by men, easy-read guides on gender-based violence, providing information and raising awareness of ID for people working with women who have been victims of gender-based violence, information services and specialist guidance on existing resources and intermediation in the public system, emotional support, legal advice); employment; avoiding overprotection (parents are usually more protective with daughters); taking into account the demands of women with ID; right to participate in decisions about their lives.
- Article 7 (children with disabilities): Six papers focus on the right of children with disabilities to express their views freely on all matters affecting them and to be provided with disability- and age-appropriate assistance to realize that right [8,46,48,67,74,75]. While most of the indicators and personal outcomes matched those already proposed and reflected in other CRPD rights, it was stressed that these must also be fulfilled during childhood and adolescence. Among these are the importance of children participating in civil life and decision-making (including health); the will and preferences of children with disabilities are respected on an equal basis with other children (e.g., children should be asked to express their views and preferences during legal procedures, using all possible ways of communication such as drawing and painting, body language, facial expressions; providing children with accessible information that allows them to express their opinion); promoting inclusion and preventing family vulnerability (e.g., avoiding separation from parents against their will on the basis of a disability and, where this is necessary, providing alternatives within the extended family or in the community in a family environment, child protection services); appropriate transition from child-specific to adult-specific services; the development of adaptive behavior skills; involving children in advocacy, decision-making or even human rights monitoring.
- Article 10 (right to life): Only two papers give measurable aspects on the right to life [76,77]. Both specifically mention providing supports to make choices about end-of-life: to express their will and preference (using a range of intentional/unintentional and formal/informal behaviors); supporters to listen to the person’s expression of preference by acknowledging, interpreting and acting on that preference); intimate or very close relationships between people with ID and their supporters (knowledge of the person’s life story; documentation of a person’s history and life story through sharing of historical stories, images and video about the person being supported, by those who had known them for a long time, across multiple areas of their life; enjoyment of their company; willingness and ability to see the person “beyond their disability”); person-centered approach (encouraging the use of supports for end-of-life care in home settings, and recognizing variations in what “home” may be like with respect to end-of-life care).
- Article 11 (situations of risk and humanitarian emergencies): States Parties shall take all necessary measures to ensure the protection and safety of persons with disabilities in situations of risk, including situations of armed conflict, humanitarian emergencies and the occurrence of natural disasters. None of the papers included in this review refer to specific indicators or personal outcomes associated with this Article.
- Article 12 (equal recognition before the law): A dozen articles [8,49,71,74,75,76,78,79,80,81] focus on the promotion of supported decision-making strategies. They specifically report on the need to replace guardianship (disapproving of legal incapacity and substituted decision-making) with the supported decision-making model: empower them to exercise their own will and preferences (training about decision-making; providing information about supported decision-making to people with disabilities, their families, and professionals; taking into consideration individual concerns, experiences with legal systems and levels of literacy; identifying communication barriers; implementing measures to enable their voice to be heard); appropriate and ongoing support to exercise decision-making capacity in all areas of their lives (person-centered planning: all forms of support in the exercise of legal capacity must be based on their specific needs, will and preferences, not on what is perceived as being in their objective best interests; support is available at nominal or no cost and lack of financial resources is not a barrier to accessing support in the exercise of legal capacity; safeguards must be established for all processes relating to legal capacity and support in exercising legal capacity); dyad of decision-maker with ID and a decision supporter (legal recognition of the support person(s) formally chosen by the person must be available and accessible, including mechanisms for third parties to verify the identity of a support person and challenge their actions if they believe that the support person is not acting in accordance with the will and preferences of the person concerned; the person has the right to refuse support and terminate or change the support relationship at any time; supporters’ respect for rights, values, goals, experiences of individual; having good interpersonal skills and the ability to recognize conflicting interest; responding to the expression of will and preference by acknowledging, interpreting and responding to it; having a close and trusting relationship with the decision-maker or the capacity to build one; using formal decision-making agreements, committing time and providing support for as long as is needed for a decision to be reached; supporting decision makers to take risks, change their minds, make decisions others may not like, and extend their experiences; helping them to access information, discussing available information in understandable ways, and advocating for decisions made to be acted on; showing commitment, familiarity with disability, good communication and advocacy skills, common sense and ethical behavior); the person is encouraged to make their own decision by providing them with a range of options but not imposing choices; the person expresses will and preference, intentionally and unintentionally, using a range of modalities (e.g., behavior, vocalization, vocal pitch, muscle tone, facial expression, eye movement, self-harm, breath, unintentional physiological functions); the meetings are set up in a comfortable environment with the consent of the person.
- Article 13 (access to justice): Only one paper specifically refers to access to justice (for women with ID who have been victims of gender-based violence) [74]. It points to the need for ways to enable them to successfully engage with the legal system; support from workers and agencies in their interactions with the justice system; supports from police and judicial officers (e.g., adjusting their language, regarding complaints and statements as serious and with the same weight as they would for persons without disabilities, taking time to explain the law and its application, recognizing them as full persons before the law); training police and judicial officers about IDD.
- Article 15 (freedom from torture or cruel, inhuman or degrading treatment or punishment): Only four papers [50,63,73,82] specify indicators or personal outcomes associated with this Article: use of seclusion or solitary confinement (i.e., restrictive interventions, exclusionary practices); restraints (physical, mechanical, chemical); harmful treatments (e.g., inappropriate sedation, forced medication, failure to meet dietary requirements).
- Article 22 (respect for privacy): The four papers [40,49,50,69] that allude to this right identify the following indicators and personal outcomes: respect for privacy by flatmates, parents and caregivers (having their own room and space: private, peace and quiet; privacy of personal and intimate information; privacy related to sexuality).

#### 3.3.6. Emotional Wellbeing

- Article 16 (freedom from exploitation, violence and abuse): 12 articles specify indicators or personal outcomes on this aspect [8,38,40,42,49,57,59,63,65,66,74,82]. Those frequently cited include freedom from concealment, abandonment, abuse or neglect; segregation or exclusion (social and physical isolation); bullying (name calling; cyberbullying) in educational and social settings; experiencing vulnerability and not feeling safe (in relationships, in immediate environment); gender-based violence; sexual abuse (being able to detect abuse), physical violence (violent relationships); economic abuse.
- Article 17 (protecting the integrity of the person): Six papers mention personal outcomes related to indicators of being treated with respect, dignity, and equity [40,47,49,51,52,63]. For professionals, these include providing unconditional support, emotional support, a listening ear, empathy, patience, and trust; and that they know the people with IDD well, understand their perspective, and value and respect them and their family. For the persons with disabilities, these include self-acceptance and self-awareness of disability, not showing low expectations of themselves because of disability; not experiencing exclusionary reactions, such as not being addressed in conversations or being ignored by professionals in different sectors; use of positive behavioral support.

#### 3.3.7. Physical Wellbeing

- Article 25 (health): 10 articles [48,50,51,56,66,67,73,83,84,85] discuss the right to health, highlighting indicators and personal outcomes associated with good physical health (e.g., healthy weight, absence of weight-related physical problems such as diabetes, gastrointestinal disorders, hypertension); prevention; access to appropriate information on health-related issues; promotion of healthy behaviors in accessible formats; good psychological health (absence of behavioral problems or psychiatric disorders); to have family and disability support worker advocacy (without conflicts of interests between their own needs/vision and those of their son or daughter); early screening and diagnosis (including comorbidities); community programs favoring cognitive, physical, and social development; shared decision-making among health care providers, children and families; supervised, justified and adjusted medication (especially, antipsychotic); rigorous data collection system and epidemiological data on prevalence of IDD and mental illness; existence of IDD mental health policy; tested for sexually transmitted diseases; no substance abuse or dependence (e.g., methamphetamine, alcohol, and marijuana).
- Article 26 (habilitation and rehabilitation): 12 papers [40,48,49,50,51,54,66,67,73,83,84,85] focus on specific elements related to habilitation and rehabilitation, such as accessing quality efficient and specialized physical and mental health care and social care (speech therapists, psychologists, psychiatrists, physiotherapists, dentists, X-ray facilities, primary and tertiary health services); appropriate and affordable early and timely health services, interventions and care; coordination and communication between health, education and social services; health services close to home; appropriate transitions between health services (e.g., from pediatric to adult services); availability, knowledge and use of assistive devices and technologies related to habilitation and rehabilitation; health staff feeling competent to care for patients with IDD (educational curricula for health professionals about disability; availability of specialized training; avoiding misperceptions; well-trained mental health professionals in dual diagnoses); health staff’s positive attitudes (looking and directly talking to person with IDD, willing to provide them with care; showing respect); individualized and capability-based services.

#### 3.3.8. Material Wellbeing

- Article 27 (work and employment): Eight papers contain employment-related aspects [39,40,42,46,47,49,59,66]: access to the labor market; having a (local) paid job (not being refused a job, promotion or interview because of disability); safe and suitable employment; satisfaction with the employment and salary (paid on an equal basis to others); adequate provision of accommodation and employment services (e.g., hours, duty, human support); employer and employee attitudes (e.g., aware of work strengths and limitations, not considering the person disadvantaged because of disability; negative attitudes when looking for employment; ongoing disability information and awareness activities for all employees; satisfactory treatment for employees); adequate job information, training and experience (advice on alternative employment; individualized training based on needs, studies, professional experience, interests and availability; skills training to find and keep a job; individual guidance to map out potential professional pathways; assessment and guidance on job options tailored to needs profile; presence of support persons to mediate with the company; tracking recruitment to make adjustments); employment as a way to meet people and friends; work-life balance measures (i.e., childcare while they work).
- Article 28 (adequate standard of living and social protection): 10 studies [8,40,43,47,53,54,59,66,67,69] specify personal outcomes or indicators associated with this Article: financial independence (adequate subsistence base); sufficient financial income to access housing (housing affordability); safe, accessible and suitable housing; aid to cover the additional expenses of supports and specialist care (economic support); having the necessary assistive products for environment and self-care (knowledge and awareness about them; customized); personal assistant when needed; leisure activities in people’s home (e.g., listening to the radio, playing instruments, being at home with friends); saving and budgeting (including for holidays); satisfaction with income; assistance with managing money and budgeting; receiving disability benefits (not losing benefits for being employed); easing bureaucracy involved in getting personal assistance and personal budgets; flexibility of support funded; provision of social assistance when needed; existence of personal budgets/personal assistance schemes and awareness of these by people with disability; stability of funding over time; strong supportive legislation.

## 4. Discussion

This systematic review sought to answer three key questions. The first was to learn about the main themes covered in publications about the CRPD and people with IDD, a broad term that combines the fields of intellectual disability (diagnosis given to individuals who meet the criteria of significant limitations both in intellectual functioning and adaptive behavior as expressed in conceptual, social, and practical skills, and is manifest before age 18) and developmental disabilities (non-categorical label for a chronic disability manifest before age 22 but limited to persons with a specific diagnosis or for those whose disability manifest before age 22 results in substantial functional limitations in three or more major life activity areas and who require long- term services and supports) [86]. The vast majority of the publications focused on one or more of the 26 CRPD Articles pertaining to specific rights (i.e., Articles 5 to 30), while only one-fifth either referred to conceptual frameworks for CRPD monitoring or proposed or applied assessment instruments. While still an emerging approach, inclusive research [34,35,36,37,38,39,40,41], which encourages people with IDD to participate as researchers at all stages of the research process, is increasingly being used or recommended in the scientific literature.

The second research question focused on identifying the conceptual frameworks used to monitor the CRPD or any of its specific Articles. All of the reviewed papers that covered this aspect underscored the relevance of the QOL framework to assess progress and identify needs and gaps in implementation. The most widely used framework was Schalock and Verdugo’s eight-domain QOL model (or a variation based on some of its specific domains, such as rights or self-determination). Furthermore, a number of specific assessment instruments developed from this model have been used in studies to explore the implementation of the CRPD (e.g., QOL-Q, Integral Scale, Gencat Scale, Personal Outcomes Scale). Other monitoring instruments used were the Rights of Persons with Disabilities Scale [11], the ITINERIS Scale [31], the National Core Indicators-Adult Consumer Survey (NCI-ACS) [3,32], and the European Child Environment Questionnaire (ECEQ). Although some studies employed qualitative methods (e.g., focus groups), quantitative questionnaires remained the most widely used and recommended approach, particularly in the case of self-report instruments.

The third research question focused on identifying indicators or personal outcome categories. The Article that received the most coverage in the reviewed papers was living independently and being included in the community (Article 19), followed by the right to freedom from exploitation, violence and abuse (Article 16), and habilitation and rehabilitation (Article 26). It is striking, however, that none of the publications referred to specific indicators associated with situations of risk and humanitarian emergencies (Article 11). In light of the global Covid-19 pandemic, this is unlikely to remain the case for much longer, and we would hope that future research will focus on the potential situations of discrimination or particular vulnerability faced by people with IDD in the fight against this pandemic (e.g., if they received appropriate information on how to prevent and treat the infection, if they received the supports they needed during the pandemic, if they were discriminated against by the health services on the grounds of their disability). Similarly, Articles 13 (access to justice) and 18 (liberty of movement and nationality) require further attention. With regard to the latter, it would be important to see studies examining the violation of the rights of people with IDD, where these people are also immigrants in a given country or refugees at a particular border.

The review of the literature and the analysis of the selected studies also revealed some limitations in the conceptual framework used, specifically with regard to the alignment of the CRPD Articles to the QOL domains. In particular, we found that Article 15 (freedom from torture or cruel, inhuman or degrading treatment or punishment), included in the rights domain by Verdugo et al. [1], was closely linked in the literature to the indicators proposed for Article 16 (freedom from exploitation, violence and abuse), assigned to the emotional wellbeing domain. Given this close relationship, and following our review of the scientific literature, we propose that Articles 15 and 16 both be included in the emotional wellbeing domain. In addition, overlaps in the indicators proposed by Lombardi et al. [2] required the removal of some repetitions (e.g., indicators for Article 30 were removed from the interpersonal relationships domain because they had also been included in the social inclusion domain; or the “safe and secure environment” indicators, which in the initial model featured as indicators of Article 14, liberty and security of the person, but also of Article 16, freedom from exploitation, violence and abuse, were finally assigned to the latter). We also reassigned indicators to a different Article where it made more sense, as a result of what we found in our literature review. For example, “dating with persons of choice” was initially included as an indicator of Article 5, equality and non-discrimination, in the rights domain. Since all of the studies included this as an indicator of Article 23, respect for home and the family, in the interpersonal relationships domain, we assigned it accordingly. In this way, as the authors themselves suggested [2], a refinement of their proposal has been made based on an exhaustive review of the literature.

Articles 6 (women with disabilities) and 7 (children with disabilities) deserve particular mention. The literature review revealed that they do not contain additional rights to those already covered in other Articles, but rather they are two cross-cutting Articles that seek to draw attention to these two specific groups in view of their particular vulnerability. Rather than including specific items in any assessment instruments that are developed, we would recommend that this information be collected as sociodemographic variables (i.e., gender, age) in order to verify whether these two conditions can have a significant impact on outcomes.

Finally, this review should be seen as a relevant, critical and necessary step in the development of future instruments to inform people with IDD of their rights, and to inform supporting professionals and family members of these rights, while at the same time to monitor the implementation of the CRPD. This review is just the first step in the process of operationalizating the CRPD through tentative definitions composed of all indicators and outcomes found in the scientific literature. That said, additional steps in each country or culture will be necessary or advisable in order to further refine the pool of indicators and personal outcomes described here, with the aim of achieving a comprehensive list that is relevant to the target group. Such an approach will help enhance the content validity of employing unique indicators to specific Articles and QOL domains. As part of this process, qualitative techniques such as focus groups and consultations with key experts and stakeholders including people with IDD are recommended in order to provide evidences of their quality and validity. Finally, their translation into specific measurable items and testing their psychometric properties will be necessary to determine their validity and utility.

## 5. Conclusions

The key points that this review highlights are several. First, the relevance of the QOL framework to assess progress and identify needs and gaps in the implementation of the CRPD. Second, the need of specific assessment instruments to explore the implementation of the CRPD. And third, the lack of studies focused on situations of risk and humanitarian emergencies, access to justice, liberty of movement and nationality, women and children with disabilities.

Although it has been more than a decade since the CRPD entered into force in a large number of countries, people with disabilities, especially people with IDD, continue to see their fundamental rights undermined. To fully implement the CRPD, there is an urgent need to operationalize its Articles through the use of a validated conceptual model, such as the QOL model discussed in this article, as a framework to develop and apply reliable and valid instruments that not only allow countries to monitor the fulfillment of the rights set out in the CRPD in the macrosystem, but especially in the microsystem and the mesosystem. It is essential that people with IDD and their support providers (natural and professional) know their rights and that these rights can be evaluated through instruments that demonstrate sufficient levels of validity and reliability. Such a tool would facilitate relating the provision of individualized supports to specific CRPD Articles and implementing evidence-based practices for people with IDD. Such a process would enhance their QOL as full citizens. Based on an analysis of the scientific literature, this study constitutes an essential first step in the operationalization of the CRPD and providing evidence of content validity for the future development of context-focused assessment instruments.

## Figures and Tables

**Table 1:** Relationship between QOL domains, QOL indicators, and CRPD Articles [adapted from [1,2]].

Domain.	CRPD Articles Based on Verdugo et al. [1]	QOL Indicators Based on Lombardi et al. [2]
Personal development	24 (education)	– Educational setting – Personal skills – Lifelong learning
Self-determination	14 (liberty and security of person)	– Freedom of movement – Freedom of choice – Personal autonomy – Safe environment – Personal control – Realizing personal goals – Secure environment
	21 (freedom of expression and opinion, access to information)	– Level of understanding the information – Using information – Opportunities to express opinion – Access to information
Interpersonal relationships	23 (respect for home and the family)	– Right to set up their own family – Right to be a parent – Dating and intimate with persons of choice
Social inclusion	8 (awareness-raising)	– Acts of awareness (e.g., projects, campaigns) to increase social inclusion
	9 (accessibility)	– Presence in cultural events – Presence in recreational or leisure events
	18 (liberty of movement and nationality)	– Physical access on community streets – Physical access to public transportation – Physical access in community buildings
	19 (living independently and being included in the community)	– Living in a home with minimum intrusion from others – Home ownership – Rental agreement
	20 (personal mobility)	– A way to be personally mobile (e.g., by walking, using a wheelchair, or using crutches) – A way to transport across environments (e.g., a car, a bike, and public transportation)
	29 (participation in political and public life)	– Membership on boards – Running for public office
	30 (participation in cultural life, recreation, leisure and sport)	– Participation and presence in cultural events (e.g., concerts, movies, theaters, and museums) – Participation in recreational or leisure events (e.g., hobbies and community activity clubs) – Opportunity to travel
Rights	5 (equality and non-discrimination)	– Presence in the community – Engagement in open employment – Participation in community activities – Dating with persons of choice
	6 (women with disabilities)	– Personalized supports – Participation in community life – Adequate financial resources
	7 (children with disabilities)	– Receiving post-natal care – Supports to enhance personal growth and development – Involved in educational program – Provision of adequate medical care – Inclusion in their family – Inclusion in the community
	10 (right to life)	– Making choices about contraception – Making choices about end-of-life decisions
	11 (situations of risk and humanitarian emergencies)	– Supplying immigrants with a disability with sufficient legal, financial, and social supports – Access to health care
	12 (equal recognition before the law)	– Access to legal services – Receiving due process – Being considered legally competent
	13 (access to justice)	– Having a defense attorney – Participation in one’s defense – Being adjudicated by a magistrate, a judge, or a jury – If guilty, receiving a fair sentence – Understanding the charge
	15 (freedom from torture or cruel, inhuman or degrading treatment or punishment)	– Personal injuries caused by others (e.g., torture and maiming) – If guilty, the punishment received is commensurate to that received by others
	22 (respect for privacy)	– Control over personal areas (e.g., bedroom, bathroom, home, or dwelling) – Personal access to communication (e.g., letters, e-mails, and phone)
Emotional wellbeing	16 (freedom from exploitation, violence and abuse)	– Living in a safe environment – Not being exploited by others (e.g., sexually, financially, socially) – Not being abused by others (e.g., physical and emotional)
	17 (protecting the integrity of the person)	– Experiencing respect – Experiencing dignity – Experiencing equality
Physical wellbeing	25 (health)	– Physical status – Nutritional status – Chronic conditions
	26 (habilitation and rehabilitation)	– Medical intervention if needed – Emotional intervention if needed – Therapy (e.g., physical, occupational, speech)
Material wellbeing	27 (work and employment)	– Full-time paid employment – Part-time paid employment – Job training programs
	28 (adequate standard of living and social protection)	– Annual income covers basic living expenses – Annual income allows for discretionary spending – Adequate housing – Unemployment insurance – Public assistance if necessary

**Table 2:** Search procedures for English and Spanish publications.

Platform	Set	Results	Search	Language
WOS	#1	39,949	TOPIC: (Convention) *OR* TOPIC: (CRPD) *OR* TOPIC: (UNCRPD) Timespan = 2008–2020 Search language = English	English
	#2	56,988	TOPIC: (“intellectual disability *”) *OR* TOPIC: (“learning disability *”) *OR* TOPIC: (“Intellectual developmental disorder*”) *OR* TOPIC: (“developmental disability *”) Timespan = 2008–2020 Search language = English	
	#3	200	#1 *AND* #2 Timespan = 2008–2020 Search language = English	
ProQuest	#1	3787	(Convención *OR* CDPCD *OR* CDPD) *AND* la.Exact(“Spanish”) Limit to: Peer reviewed, Date: After December 31, 2007	Spanish
	#2	404	(“discapacidad intelectual” *OR* “trastorno* del desarrollo” *OR* “discapacidad* del desarrollo”) *AND* la.Exact(“Spanish”) Limit to: Peer reviewed, Date: After December 31, 2007	
	#3	52	**1***AND***2** Limit to: Peer reviewed, Date: After December 31, 2007	
Scopus	#1	64	(TITLE-ABS-KEY (convención) *OR* TITLE-ABS-KEY (cdpd) *OR* TITLE-ABS-KEY (cdpcd)) *AND* PUBYEAR > 2007 *AND* (LIMIT-TO (LANGUAGE, “Spanish”))	Spanish
	#2	216	(TITLE-ABS-KEY (“discapacidad intelectual”) *OR* TITLE-ABS-KEY (“discapacidad* del desarrollo”) *OR* TITLE-ABS-KEY (“Trastorno* del desarrollo”)) *AND* PUBYEAR > 2007 *AND* (LIMIT-TO (LANGUAGE, “Spanish”))	
	#3	1	**1** *AND* **2**	
**N total**		253		

**Table 3:** Proposed theoretical frameworks for CRPD monitoring.

Ref	Framework	Proposed/Applied Instruments	Main CRPD Article(s) *	Country
[11]	Quality of life and self-determination	Quality of Life Questionnaire (QOL-Q) AIR Self-Determination Scale Rights of Persons with Disabilities	35	Nepal, Zambia, United States
[28]	Quality of life (satisfaction)	Personal Wellbeing Index - intellectual disability	19, 35	Australia
[29]	Quality of life (educational)	---	7, 24, 35	Australia
[8]	Quality of life	Integral Scale Gencat Scale	6, 12, 19, 23, 24, 29, 35	Spain
[1]	Quality of life	Integral Scale Gencat Scale Personal Outcomes Scale	35	United States, Spain
[2]	Quality of life	---	35	Brazil, Canada, Czech Republic, Germany, Italy, Israel, Portugal, Spain, Taiwan, United States
[9]	Quality of life	---	31, 32	United Kingdom
[30]	Quality of life	Focus groups and interviews	7, 21, 35	Australia

* Main CRPD Article(s) addressed in the publication.

**Table 4:** Instruments applied or proposed in the studies and their relationship to CRPD Articles and the quality model.

Ref	Instrument(s)	Target Population	Respondents	Construct(s) Assessed	N Items	Related CRPD Article(s)	Region
[33]	European Child Environment Questionnaire (ECEQ)	Children (8–12 years old) with cerebral palsy	Report of others: relatives	Physical environment, Social support, Attitudes	51	9, 18, 21	Europe
[31]	ITINERIS Scale	Adults with ID	Self-report	Rights	30	Preamble 1–30	South America
[32]	National Core Indicators-Adult Consumer Survey (NCI-ACS)	Adults aged 18 or over with IDD	Self-report	Quality of services	35	3, 8, 9, 14, 19, 21, 22, 23, 24, 25, 27, 30	United States
[3]	NCI-ACS				16	3, 8, 14, 21, 22, 23, 27	United States
[13]	Gencat Scale	Adults aged 18 or over with ID	Report of others: professionals	Rights (within the framework of QOL)	10	5, 12, 16, 17, 22, 29	Spain
[13]	Integral Scale (Gómez et al., 2011)		Self-report		8		
